# Effects of Melatonin on the Transcriptome of Human Granulosa Cells, Fertilization and Blastocyst Formation

**DOI:** 10.3390/ijms23126731

**Published:** 2022-06-16

**Authors:** Isao Tamura, Hiroshi Tamura, Mai Kawamoto-Jozaki, Yuichiro Shirafuta, Taishi Fujimura, Yumiko Doi-Tanaka, Yumiko Mihara, Toshiaki Taketani, Norihiro Sugino

**Affiliations:** Department of Obstetrics and Gynecology, Graduate School of Medicine, Yamaguchi University, Minamikogushi 1-1-1, Ube 755-8505, Japan; isao@yamaguchi-u.ac.jp (I.T.); mkawa@yamaguchi-u.ac.jp (M.K.-J.); yshirafu@yamaguchi-u.ac.jp (Y.S.); taishif0122@gmail.com (T.F.); ydoi@yamaguchi-u.ac.jp (Y.D.-T.); andy-yu@yamaguchi-u.ac.jp (Y.M.); taketani@yamaguchi-u.ac.jp (T.T.); sugino@yamaguchi-u.ac.jp (N.S.)

**Keywords:** melatonin, ART, granulosa cells, transcriptome

## Abstract

Melatonin is a promising reagent that can improve assisted reproductive technology (ART) outcomes in infertility patients. However, melatonin is not effective for all infertile patients, and it remains unclear for which patients melatonin would be effective. This study examined the effects of melatonin on ART outcomes and examined its mechanisms. Melatonin increased the fertilization rate in patients whose fertilization rates in the previous cycle were less than 50%, but not in patients whose fertilization rates were more than 50% in the previous cycle. Melatonin increased the blastocyst formation rate in patients whose embryo development rates in the previous cycle were less than 50%, but not in patients whose embryo development rates were more than 50% in the previous cycle. To clarify its mechanisms, transcriptome changes by melatonin treatment in granulosa cells (GCs) of the patients were examined by RNA-sequence. Melatonin treatment altered the transcriptomes of GCs of patients with poor ART outcomes so that they were similar to the transcriptomes of patients with good ART outcomes. The altered genes were associated with the inhibition of cell death and T-cell activity, and the activation of steroidogenesis and angiogenesis. Melatonin treatment was effective for patients with poor fertilization rates and poor embryo development rates in the previous ART cycle. Melatonin alters the GCs transcriptome and, thus, their functions, and this could improve the oocyte quality, leading to good ART outcomes.

## 1. Introduction

Melatonin (N-acetyl-5-methoxytryptamine) is a hormone secreted by the pineal gland in a circadian manner. Its secretion is regulated by light and dark environments, and it functions in the regulation of circadian rhythms and sleep [1]. Melatonin is a direct free radical scavenger with more powerful antioxidant potential than conventional antioxidants such as vitamins C and E, mannitol, and glutathione [2]. In recent years, remarkable advances have been made in assisted reproductive technology (ART) for infertility treatment [3,4]. However, satisfactory conception rates have not been achieved. The major cause of this is attributed to problems in the quality of oocytes [5]. It is thought that oxidative stress caused by reactive oxygen species (ROS) in the follicle is one of the critical factors determining oocyte quality, because oocytes under high oxidative stress show poor oocyte quality with poor fertilization ability and poor embryo development [6,7]. We previously demonstrated that melatonin protects oocytes and granulosa cells (GCs) from apoptosis by scavenging ROS produced within follicles during ovulation [6,8,9,10,11]. Our previous clinical study showed that oral melatonin administration for infertile women increases intrafollicular melatonin concentrations, reduces intrafollicular oxidative stress, and elevates fertilization and pregnancy rates by ART [11]. After our initial study, other reports also showed that melatonin administration improves the clinical outcome of ART as shown by increases in the number of mature oocytes, the fertilization rate, and the number of high-quality embryos [12,13,14,15,16]. Therefore, melatonin is a promising reagent that can improve ART outcomes by reducing intrafollicular oxidative stress. As a result, we have used melatonin to treat a number of patients who failed to become pregnant by ART. However, while melatonin treatment improved the fertilization or embryo development in some cases, it had no effect in other cases. In other words, melatonin is not effective for all infertile patients, and it remains unclear for which patients melatonin would be effective. Therefore, it is necessary to identify the characteristics of patients whose ART results may be improved by melatonin treatment.

The mechanisms by which melatonin improves oocyte quality have not been well clarified. The quality and development potential of oocytes are linked to the status of GCs [17,18]. Because melatonin protects GCs from apoptosis by scavenging ROS, melatonin may improve oocyte quality by altering the cellular functions of GCs. To investigate the altered cellular functions, it is important to examine the effects of melatonin administration on gene expressions in GCs. Although previous reports showed that melatonin alters the gene expression of human GCs, these studies were carried out only in vitro culture of GCs [19,20,21]. Therefore, it remains unclear whether oral melatonin administration alters gene expression of human GCs in vivo. This led us to examine the effects of melatonin on the transcriptome of GCs, which we expected would clarify how melatonin treatment improves ART outcomes.

In this study, we retrospectively examined patients whose ART outcomes were improved by melatonin treatment. Furthermore, to clarify its mechanisms, we obtained GCs in the ART cycle before and during melatonin treatment from the same patients and analyzed the transcriptome changes by RNA-sequence.

## 2. Results

### 2.1. Association between Previous Fertilization Rate and the Effect of Melatonin

**Figure 1:**
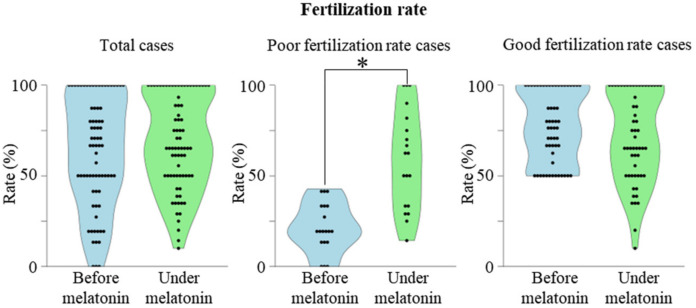
Association between previous fertilization rate and the effect of melatonin. Effect of melatonin on the fertilization rate. The fertilization rates in the cycles before and under melatonin treatment are shown in the violin plot. Left graph: Effect of melatonin in total 75 cases. Middle graph: Effect of melatonin in 19 cases whose fertilization rate before melatonin treatment was less than 50% (Poor fertilization rate cases). Right graph: Effect of melatonin in 56 cases whose fertilization rate before melatonin treatment was more than 50% (Good fertilization rate cases). * *p* < 0.01 vs. before melatonin.

To retrospectively evaluate the effects of melatonin on fertilization rates, the fertilization rates of 75 women were compared between the cycle before melatonin treatment and the cycle under melatonin treatment. Overall, fertilization rates were not significantly different before and under melatonin treatment (Figure 1, left graph). We divided the patients into two groups according to their fertilization rate before melatonin treatment; 19 patients whose fertilization rate before melatonin treatment was less than 50% (Poor fertilization rate cases), and 56 patients whose fertilization rate before melatonin treatment was more than 50% (Good fertilization rate cases). The backgrounds of the two groups (Table 1) were not significantly different.

Then, the effect of melatonin on the fertilization rate was compared, respectively. Melatonin significantly improved the fertilization rate in poor fertilization rate cases (Figure 1, middle graph), but no further improvement was observed in good fertilization rate cases (Figure 1, right graph).

### 2.2. Association between Previous Embryo Development Rate (EDR) and the Effect of Melatonin

**Figure 2:**
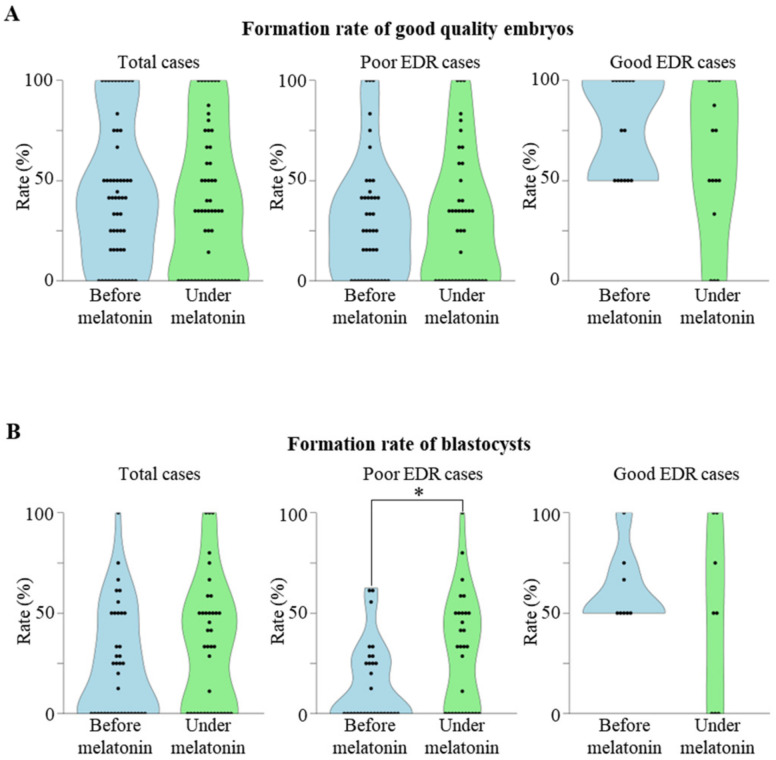
Association between previous embryo development rate (EDR) and the effect of melatonin. (**A**) Effect of melatonin on the formation rate of good quality embryos. The formation rates of good quality embryos in the cycles before and under melatonin treatment are shown in the violin plot. Left graph: Effect of melatonin in total 56 cases. Middle graph: Effect of melatonin in 41 cases with poor embryo development rate (EDR) (either the formation rate of good quality cleavage-stage embryos or the formation rate of blastocysts before melatonin treatment was less than 50%). Right graph: Effect of melatonin in 15 cases with good EDR. (**B**) Effect of melatonin on the formation rate of blastocysts. The formation rates of blastocysts in the cycles before and under melatonin treatment are shown in the violin plot. Left graph: Effect of melatonin in total 38 cases. Middle graph: Effect of melatonin in 30 cases with poor EDR. Right graph: Effect of melatonin in 8 cases with good EDR. * *p* < 0.05 vs. before melatonin.

We next evaluated the effect of melatonin on embryo development in the 56 cases with good fertilization rate, because they had a sufficient number of fertilized oocytes to analyze the subsequent embryo development. Overall, the formation rate of good quality embryos was not significantly different before and under melatonin treatment (Figure 2A, left graphs). We divided the patients into two groups according to their EDR before melatonin treatment; 41 cases with poor EDR (either the formation rate of good quality cleavage-stage embryo or the formation rate of blastocysts was less than 50% before melatonin treatment), and 15 cases with good EDR. The backgrounds of the two groups (Table 1) were not significantly different. The formation rate of good quality embryos was not significantly different before and under melatonin treatment in both good and poor EDR cases (Figure 2A, middle and right graphs).

Because the developmental capacity of embryos is more associated with the formation of blastocysts than with the quality of cleavage-stage embryos [22,23,24], we evaluated the effect of melatonin on the blastocyst formation rate. Because 18 of the 56 cases underwent embryo transfer at the cleavage-stage and had no embryos to be cultured until the blastocyst stage, they were excluded from the analysis. In total, the blastocyst formation rate was evaluated in 38 cases. Overall, the blastocyst formation rate was not significantly different before and under melatonin treatment (Figure 2B, left graph). Melatonin significantly improved the blastocyst formation rate in poor EDR cases (Figure 2B, middle graph), but no further improvement was observed in good EDR cases (Figure 2B, right graph).

We evaluated the implantation rate and pregnancy rate after melatonin treatment. In poor fertilization rate cases, 12 cases (22 embryos) underwent embryo transfer in the cycle during the melatonin treatment and 1 case became pregnant (implantation rate; 4.5%, pregnancy rate; 8.3%). In poor EDR cases, 25 cases (38 embryos) underwent embryo transfer in the cycle during the melatonin treatment and 4 cases became pregnant (implantation rate; 10.5%, pregnancy rate; 16.0%). In good EDR cases, 11 cases (16 embryos) underwent embryo transfer in the cycle during the melatonin treatment and 1 case became pregnant (implantation rate; 6.3%, pregnancy rate; 9.1%). There were no significant differences in the implantation rate and pregnancy rate between three groups. Therefore, it was indicated that implantation rate and pregnancy rate were not associated with the improvement in fertilization or blastocyst formation rate in this study.

### 2.3. Effect of Melatonin Treatment on Transcriptome Profile in GCs

**Figure 3:**
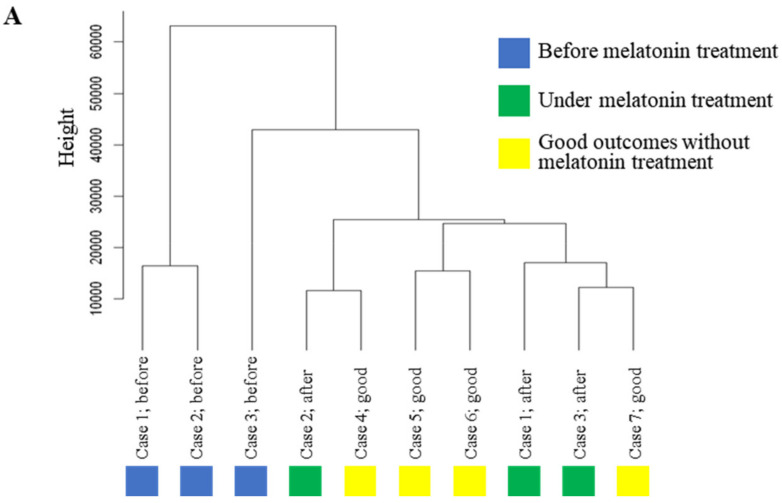

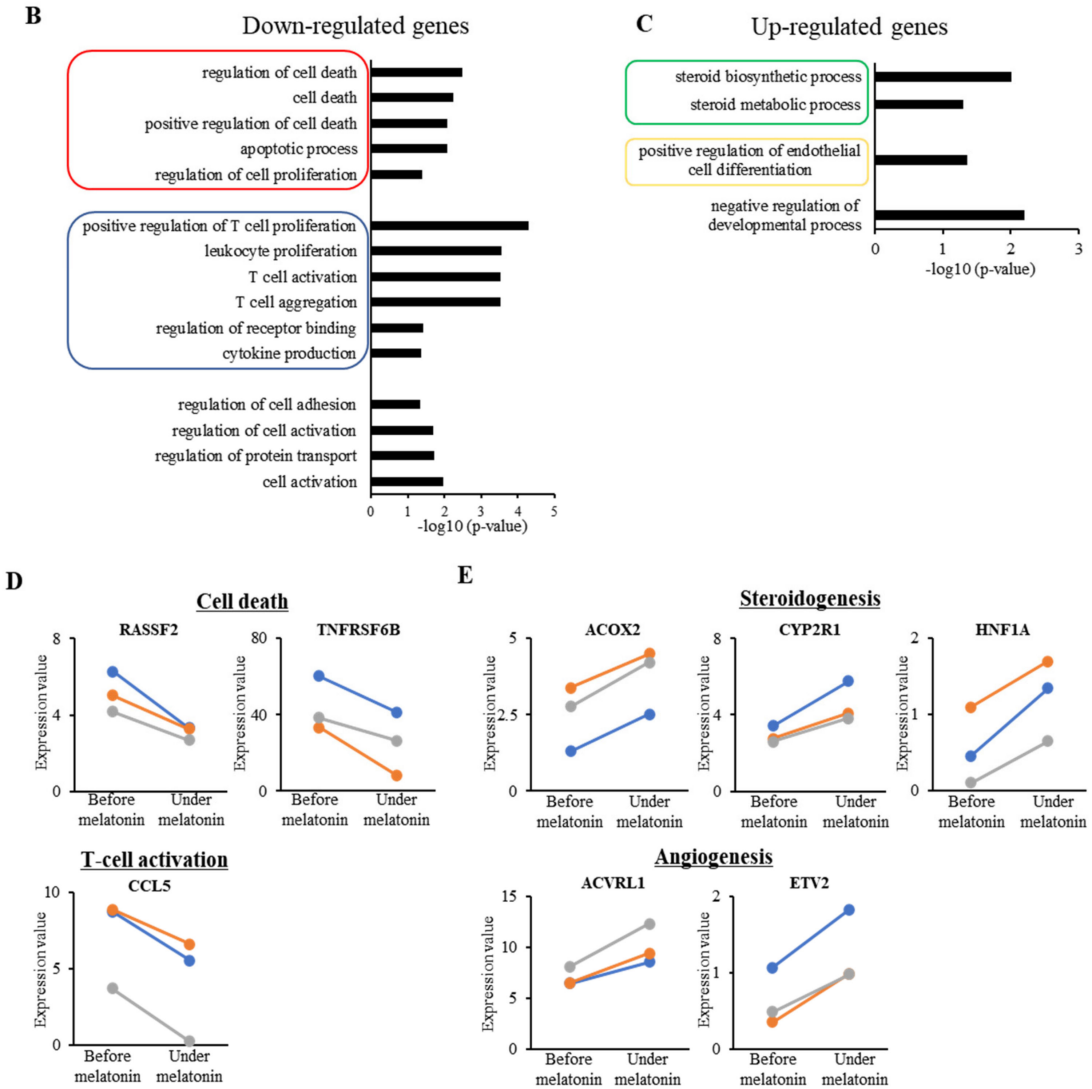
Effect of melatonin treatment on transcriptome profile in GCs. (**A**) Hierarchical cluster analysis comparing the transcriptome in GCs. Each color indicates the cases before melatonin treatment (Cases No. 1-3, blue), under melatonin treatment (Cases No. 1-3, green) and the cases with good ART outcomes without melatonin treatment (Cases No. 4-7, yellow). Distances of gene expression pattern are indicated as height. (**B**) Enriched gene ontology terms and *p*-values in down-regulated genes by melatonin treatment. The terms circled in red are associated with cell death, and the terms circled in blue are associated with T-cell activation. (**C**) Enriched gene ontology terms and *p*-values in up-regulated genes by melatonin treatment. The terms circled in green are associated with steroidogenesis, and the terms circled in yellow are associated with angiogenesis. (**D**) Changes in mRNA expression level of the representative down-regulated genes by melatonin treatment. Expression values before and under melatonin treatment are shown as dot plots of TPM determined by RNA-sequence. Blue line and dots; Case 1. Orange line and dots; Case 2. Gray line and dots; Case 3. Gene names and associated cellular functions are shown above the graphs. (**E**) Changes in mRNA expression level of the representative up-regulated genes by melatonin treatment. Expression values before and under melatonin treatment are shown as dot plots of TPM determined by RNA-sequence. Blue line and dots; Case 1. Orange line and dots; Case 2. Gray line and dots; Case 3. Gene symbols and associated cellular functions are shown above the graphs.

For this experiment, we obtained GCs in the cycles before and during melatonin treatment from three patients whose blastocyst formation was significantly improved by melatonin treatment (Table 2, Cases No. 1–3). For controls, we obtained GCs from four patients whose blastocyst formation rate was more than 50% and who became pregnant without melatonin treatment (Table 2, Cases No. 4–7). These 10 samples were subjected to RNA-sequence analysis. As shown in Figure 3A, a hierarchical clustering analysis showed that GCs before melatonin treatment (Cases No. 1–3; indicated by blue boxes) were classified into different clusters of those with good outcomes without melatonin treatment (Cases No. 4–7; indicated by yellow boxes). GCs under the melatonin treatment (Cases No. 1–3; indicated by green boxes) were classified into the same clusters as GCs with good outcomes without melatonin treatment. These results suggest that melatonin treatment altered the transcriptomes of GCs of patients with poor ART outcomes so they were similar to the transcriptomes of patients with good ART outcomes.

Melatonin treatment down-regulated 97 genes and up-regulated 152 genes (Table S1). The top 20 down- or up-regulated genes according to the *p*-values are defined as the top differentially expressed genes (DEGs) (Table 3). The expression levels of several top DEGs (*COX7B*, *CXCL*, *TRAF6* and *CENPV*) were examined by real time RT-PCR, which validated that melatonin treatment significantly altered their expression levels (Figure S1). According to a gene ontology analysis, the down-regulated genes were associated with cell death (circled in red) and T-cell activation (circled in blue) (Figure 3B, Table S2), while the up-regulated genes were associated with steroidogenesis (circled in green) and angiogenesis (circled in yellow) (Figure 3C, Table S2). The changes in mRNA expression level of the representative genes in each function are shown in Figure 3D,E. Although we performed a pathway analysis, there were no enriched terms in up- or down-regulated genes.

## 3. Discussion

This study revealed that melatonin treatment is effective for patients who had a poor fertilization rate or poor embryo development rate (EDR) in the previous ART cycle. Furthermore, this is the first study to show that melatonin treatment alters the transcriptome in GCs, which may contribute to improving ART outcomes.

We and others previously reported the favorable effects of melatonin administration on ART outcomes [11,12,13,14,15,16]. However, melatonin is not always effective for all patients, and it remains unclear for which patients melatonin would be effective. The present results reveal that melatonin increases the fertilization rate in patients whose fertilization rates in the previous cycle were less than 50%, but not in patients whose fertilization rates were more than 50% in the previous cycle. In addition, melatonin increases the blastocyst formation rate in patients whose EDR in the previous cycle was less than 50%, but not in patients whose EDR was more than 50% in the previous cycle. ART outcomes are closely associated with oocyte quality. One of critical factors determining oocyte quality is oxidative stress caused by ROS in the follicle, because excessive ROS is cytotoxic to oocytes and GCs [6,25]. In fact, the oocytes retrieved from the follicles under high oxidative stress show poor quality, and poor ability of fertilization and embryo development [7,11]. We previously reported that melatonin administration is effective in decreasing oxidative stress and improving oocyte quality [11]. Therefore, it is likely that the cases with poor fertilization rate or poor EDR were under oxidative stress and that melatonin treatment could improve ART outcomes by scavenging ROS. These facts appear to explain why melatonin treatment was effective only for patients with poor fertilization rate and poor EDR. In fact, we found that melatonin has protective effects on mouse GCs in the presence of oxidative stress, but not in its absence [26]. Taken together, our results provide important insights on the selection of patients for melatonin treatment. Although melatonin treatment improved the blastocyst formation rate, the formation rate of good quality cleavage stage embryos was not affected. This is not surprising because the development capacity of embryos is more closely associated with blastocyst formation than with the quality of cleavage-stage embryos [22,23]. Only 30% of cleavage-stage embryos can reach the blastocyst stage [24]. These facts indicate that the developmental capacity of embryos should be determined by monitoring the developmental process up to the blastocyst stage. Therefore, it is reasonable to conclude that melatonin improves embryo development because melatonin increased the blastocyst formation rate.

It is well known that GCs are involved in the regulation of oocyte quality [17,18]. Because melatonin protects GCs from apoptosis by scavenging ROS [6,8,9,10,11], it may improve oocyte quality by altering the cellular functions of GCs. Therefore, it is important to examine whether melatonin treatment alters the gene expressions of GCs. Several studies have shown that supplementing culture medium with melatonin alters the gene expressions of *VEGF*, *StAR* and *CYP19A1* in human GCs, which were collected at oocyte retrieval [20,21,27]. However, these GCs no longer exist in the follicles and are not associated with oocytes. Therefore, it is unclear whether these in vitro effects of melatonin are also true in GCs within the follicles. While melatonin administration in vivo has previously been shown to affect gene expressions in mouse GCs, such as *Sirt1* and *Myo10* [28,29], the present study is the first to show it also affects the transcriptome in human GCs. Our analysis revealed that melatonin administration altered the transcriptome in human GCs of patients with poor ART outcomes into that of patients with good ART outcomes, indicating that melatonin improves ART outcomes by altering the expressions of a number of genes in human GCs. In other words, melatonin improves the cellular functions of GCs by reducing oxidative stress.

Gene ontology analysis revealed that one of the cellular functions associated with down-regulated genes by melatonin treatment was cell death, including the genes such as *RASSF2* and *TNFRSF6B* (Figure 3D). These genes are associated with cell death because their expressions increase under oxidative stress, which induces apoptosis [30,31,32]. These results are consistent with the previously reported function of melatonin in protecting GCs from apoptosis by scavenging ROS [6,8,9,10,11]. Another cellular function associated with down-regulated genes was T-cell activation, including the gene *CCL5* (Figure 3D). The effect of melatonin on immunomodulation has not been well clarified. *CCL5* is induced by oxidative stress and is involved in the chemotaxis and activation of T lymphocytes [33]. This suggests that by decreasing *CCL5* expression in GCs, melatonin suppresses the accumulation of activated T lymphocytes around GCs. In fact, melatonin suppresses both oxidative stress-induced *CCL5* expression in bovine mammary epithelial cells [34] and the chemotaxis of leukocytes [35]. Given that apoptosis is induced by the accumulation of cytotoxic T-cells [36], melatonin may suppress the cellular function of GCs that activates T-cells, which contributes to the suppression of GC apoptosis.

Among the genes up-regulated by melatonin, some seem to be associated with angiogenesis. Angiogenic factors secreted by GCs play essential roles in angiogenesis during ovulation [37,38]. We reported that the expression of vascular endothelial growth factor (*Vegf*) increases after the LH surge in rat GCs [39]. The vascularization status of ovarian follicles affects the quality of oocytes by regulating the oxygen supply. An adequate blood supply increases the intrafollicular oxygen levels [40]. A decrease in the intrafollicular oxygen level due to poor vascularization at the perifollicular region causes metabolic disorders and chromosomal abnormalities in oocytes, leading to poor oocyte quality [40,41]. Therefore, angiogenesis is an essential event determining oocyte quality. In fact, the concentrations of angiogenic factors, including VEGF, are higher in follicular fluid with mature oocytes than in that with immature oocytes [42,43]. In addition, oocytes from the follicle with higher perifollicular vascularity show higher rates of fertilization, embryo development and implantation than those with poor vascularity [44,45]. Therefore, it is likely that melatonin improves the angiogenetic function of GCs, which contributes to improving oocyte quality. In fact, melatonin increases *VEGF* expression in cultured human GCs, supporting the involvement of melatonin in angiogenesis [20].

Another cellular function associated with up-regulated genes is steroidogenesis. The relationship between melatonin and steroidogenesis has been reported in cultured human GCs [19,21,27]. We previously reported that the cytoprotective effects of melatonin as an antioxidant are essential for progesterone production in human GCs [46]. The present study found that melatonin up-regulated the genes associated with steroidogenesis, such as *ACOX2*, *CYP2R1* and *HNF1A* (Figure 3D). These genes are involved in the metabolism of cholesterol [47,48,49], which is a precursor of progesterone. Progesterone production from GCs is suggested to be important for oocyte maturation during ovulation [50]. Therefore, it is likely that melatonin treatment improves progesterone production in GCs during ovulation, which may improve oocyte quality.

In addition to the role of melatonin as a direct radical scavenger, melatonin can affect gene expressions via the membrane melatonin receptors (MT1 and MT2), both of which are expressed in GCs and oocytes [51,52]. Therefore, there is a possibility that melatonin alters the cellular functions of GCs by activating the signaling pathway downstream of melatonin receptors. However, considering that melatonin treatment is effective for patients with poor fertilization or poor EDR, and that their oocytes are under higher oxidative stress [7,11], it is more likely that melatonin improves the cellular functions of GCs by scavenging ROS.

In conclusion, the present study showed that melatonin treatment is effective for patients with poor fertilization rate and poor ER in the previous ART cycle. This will help to identify patients that would most benefit from melatonin treatment. Melatonin reduces oxidative stress and protects GCs in patients with poor ART outcomes. This improves the cellular functions of GCs by altering the transcriptomes of GCs, which may contribute to improving oocyte quality, leading to good ART outcomes.

## 4. Materials and Methods

### 4.1. Study Population

This retrospective study included 75 female patients who had previously failed to become pregnant by conventional in vitro fertilization (cIVF) or intracytoplasmic sperm injection (ICSI), followed by embryo transfer at Yamaguchi University Hospital. They were treated with melatonin in the next ART cycle. Informed consent was obtained from all the patients in this study. The study design was reviewed and approved by the institutional review board of Yamaguchi University Hospital.

### 4.2. ART Procedure

Controlled ovarian hyperstimulation (COH) was performed using standard gonadotropin releasing hormone agonist (GnRHa)/follicle-stimulating hormone (FSH) protocols, as we reported previously [53]. Nasal spray GnRHa (900 μg/day) was given from the mid-luteal phase in the previous cycle to continuously suppress pituitary gonadotropin secretion until the injection of HCG (10,000 IU). COH was initiated from the 2nd day of the IVF-ET cycle by the injection of 225 IU FSH for 3 days, followed by a daily injection of 150 IU HMG. When more than three leading follicles reached 18 mm or more, HCG was injected for ovulation induction. Oocyte retrieval was carried out 35 h after HCG injection. For patients who showed poor response to COH in the previous IVF cycle, they underwent short GnRH agonist protocol. In this protocol, COH and GnRHa were simultaneously initiated from the 2nd day of the IVF-ET cycle. 300 IU FSH or 300 IU HMG was daily injected until the day of HCG injection. The semen samples were collected by masturbation and the motile sperm was collected by the swim-up technique. Oocytes were fertilized by either cIVF or ICSI with Piezo-assisted ICSI system (Prime Tech Ltd., Tokyo, Japan). If the sperm concentration was low (motile sperm concentration is less than 5 × 10^6^/mL after swim-up) or there was a history of fertilization failure (previous fertilization rate under cIVF was less than 25%), all retrieved oocytes were fertilized by ICSI. Fertilization was confirmed on day 1 (17–19 h after insemination) by the presence of two pronuclei. The quality of the cleavage-stage embryo was assessed on day 2 according to Veeck’s criteria. Grade 1 and grade 2 embryos were defined as good quality embryos, as reported previously [54,55]. Blastocyst formation was assessed on day 5. A fresh embryo was transferred on either day 2 (cleavage stage) or day 5 (blastocyst stage).

### 4.3. Melatonin Treatment

Patients who failed to become pregnant in the previous ART cycle due to poor fertilization rate, poor embryo development rate (EDR) (low formation rate of the good quality embryo or low formation rate of blastocyst), or repeated ART failure were administered melatonin orally in the next cycle, as reported previously [11]. They were given a 3 mg tablet of melatonin orally at 22:00 h from the fifth day of the previous menstrual cycle until the day of oocyte retrieval. For patients who were treated with a short GnRH agonist protocol, melatonin was simultaneously started on day 1 of COH.

### 4.4. Outcomes

The numbers of retrieved oocytes, fertilized oocytes, cleavage-stage embryos, and their quality, and blastocysts were evaluated in each cycle. Fertilization rate was expressed as the ratio of the number of fertilized oocytes to the number of retrieved oocytes. The formation rates of good quality embryos and blastocysts were expressed as a ratio of the number of good quality embryos (grade1 and grade 2) or blastocysts to the number of fertilized oocytes. If the embryo was transferred at the cleavage stage, the blastocyst formation rate was expressed as the ratio of the number of blastocysts to the number of fertilized oocytes minus the number of transferred embryos. Cases where embryos were transferred at the cleavage stage were excluded from the analysis of blastocyst formation rate. Outcomes of the cycles before and during melatonin treatment were compared. Cases in which different fertilization methods (cIVF vs. ICSI) were used between the cycles before and under melatonin treatment were excluded from the analysis. Implantation rate was expressed as the ratio of the number of gestational sacs to the number of transferred embryos. Pregnancy rate was expressed as the ratio of the number of pregnant cases to the number of cases who underwent fresh embryo transfer.

### 4.5. Transcriptome Analysis of Human GCs

Human GCs obtained from seven patients at oocyte retrieval were used for the transcriptome analysis. For three of the seven patients, in which blastocyst formation was not observed before melatonin treatment but was significantly improved under melatonin treatment, GCs were obtained in the cycles before and under melatonin treatment. The other four patients had good ART outcomes (their blastocyst formation rate was more than 50% and they became pregnant by the transfer of these embryos). Their GCs were used as controls of good ART outcomes. The ages and blastocyst formation rates of the seven patients are shown in Table S1. GCs were collected from follicular fluids of IVF-ET patients as reported previously [46]. After the removal of the oocyte–cumulus complex, cells were carefully aspirated from the follicular fluid and subsequently separated from red blood cells with Ficoll-Paque (Amersham Pharmacia Biotech AB, Uppsala, Sweden) by centrifuging at 500× *g* for 20 min. Total RNA was isolated from the cultured cells with a RNeasy^®^ Mini Kit (Qiagen, Valencia, CA, USA). In total, 10 samples (3 before-melatonin treated GCs, 3 under-melatonin treated GCs, and 4 control GCs) were subjected to RNA-sequence analysis, as reported previously [56,57,58]. mRNA was purified with oligo dT beads (NEBNext Poly (A) mRNA magnet Isolation Module, New England Biolabs, NEB, Beverly, MA, USA). Complementary DNA (cDNA) libraries for Illumina sequencing were generated with NEBNext Ultra II RNA library Prep kit (NEB) and NEBNextplex Oligos for Illumina. The confirmed libraries were sequenced with an Illumina Next-seq DNA sequencer with a 75-bp pair-end cycle sequencing kit (Illumina, San Diego, CA, USA). To produce the raq bcl, or base call files, quality assessment and image analyses were performed using Next-seq packaging software Real Time Analysis, and bcl2fastaq Conversion Software v2.19 (Illumina) was used for de-multiplexing the samples. Reads with more than two ambiguous nucleotides and reads with quality scores less than 20 as calculated by the Phred program were removed using CLC Genomics Workbench software (ver. 8.01, Qiagen, Valencia, CA, USA). Long reads with more than 1000 nucleotides and short reads with fewer than 20 nucleotides were also discarded. Trimmed reads were mapped to the human reference genome GRCh37 in default settings. The numbers of the identified transcript and sequenced paired end reads in each sample are shown in Table S3. Clustering analyses were performed using the R package “Cluster” [59]. Gene expression values were calculated as “transcripts per million” (TPM). We added 0.5 to the TPM value before the following calculation. Genes whose TPM increased more than 1.4-fold by melatonin treatment and whose *p*-values were < 0.05 by paired *t*-test were defined as genes that were up-regulated by melatonin treatment. Similarly, genes whose TPM decreased more than 1.4-fold by melatonin treatment and whose *p*-values were < 0.05 were defined as genes that were down-regulated by melatonin treatment. The top 20 down- or up-regulated genes according to the *p*-values are defined as the top differentially expressed genes (DEGs) (Table 3). Gene ontology was analyzed with DAVID Bioinformatics Resources v.6.8 to determine the functions of these genes [60]. Gene ontology terms with *p*-values < 0.05 were considered as enriched terms. These terms were summarized by removing redundancy and plotted using reduce and visualize gene ontology (REVIGO) with Allowed Similarity as “medium (0.5)” [61,62].

### 4.6. Real-Time RT-PCR

The relative mRNA expressions of several top DEGs (*COX7B*, *CXCL*, *TRAF6* and *CENPV*) were examined by real-time RT-PCR as reported previously [56] with sequence-specific primer sets (Table S4). *GAPDH* was used as an internal control.

### 4.7. Statistical Analysis

Mann–Whitney U test or Chi-squared test was applied to analyze the difference in backgrounds of the two groups. Wilcoxon’s rank-sum test was used to evaluate the differences in ART outcomes before and under melatonin treatment. All statistical analyses were performed using SPSS for Windows version 11 (SPSS Inc., Chicago, IL, USA). Differences were considered significant at *p*-values < 0.05.

## Figures and Tables

**Table 1:** Backgrounds of patients.

	Poor Fertilization Rate Cases	Good Fertilization Rate Cases	Poor EDR Cases	Good EDR Cases
No. of patients	19	56	41	15
Patient age (mean ± SD)	35.4± 3.7	37.3 ± 4.3	36.9 ± 4.2	38.3 ± 4.1
Patient’s complications				
Myoma/adenomyosis (%)	15.8	16.1	26.7	12.2
Endometriosis (%)	26.3	16.1	13.3	17.1
PCOS (%)	5.3	5.4	0.0	7.3
Tubal factor (%)	21.1	21.4	13.3	24.4
Unilateral obstruction (%)	10.5	12.5	13.3	12.2
Bilateral obstruction (%)	10.5	8.9	0.0	12.2
Male factor (%)	15.8	16.1	26.7	12.2
Asthenozoospermia (%)	5.3	8.9	13.3	7.3
Oligospermia (%)	10.5	7.1	13.3	4.9
EDR, embryo development rate				

**Table 2:** Information on cases provided for transcriptome analysis.

		Blastocyst Formation Rate (%)		GnRH Agonist Protocol		Fertilization	
Case No.	Age	Before Melatonin Treatment	Under Melatonin Treatment	Before Melatonin Treatment	Under Melatonin Treatment	Before Melatonin Treatment	Under Melatonin Treatment
1	32	0	40.0	long protocol	long protocol	ICSI	ICSI
2	33	0	28.9	long protocol	long protocol	cIVF	cIVF
3	36	0	100.0	long protocol	long protocol	ICSI	ICSI
4	35	61.5		long protocol		cIVF	
5	36	50.0		long protocol		cIVF	
6	34	50.0		short protocol		cIVF	
7	37	100.0		long protocol		cIVF	
long protocol, GnRHa (900 μg/day) was given from the mid-luteal phase in the previous cycle until two days before oocyte retrieval.							
cIVF, conventional in vitro fertilization			ICSI, intracytoplasmic sperm injection				

**Table 3:** List of top DEGs.

Down-Regulated Genes		Up-Regulated Genes	
Gene	*p* Value	Gene	*p* Value
*NUP62CL*	1.08 × 10^−03^	*RP11-211N8.3*	3.88 × 10^−04^
*TRAF6*	1.40 × 10^−03^	*SNRPGP2*	9.07 × 10^−04^
*ZNF501*	1.98 × 10^−03^	*RPS17P5*	9.07 × 10^−04^
*RP11-355I22.7*	2.26 × 10^−03^	*RPS11P7*	9.07 × 10^−04^
*RP11-3N2.13*	3.29 × 10^−03^	*CTD-2013M15.1*	9.94 × 10^−04^
*ZDHHC15*	5.43 × 10^−03^	*COX7B*	1.06 × 10^−03^
*CENPV*	5.82 × 10^−03^	*RP4-738P15.1*	1.36 × 10^−03^
*RP11-564C4.6*	6.78 × 10^−03^	*RP11-787I22.3*	1.38 × 10^−03^
*CD244*	6.83 × 10^−03^	*RP11-874J12.4*	1.39 × 10^−03^
*AC078883.3*	7.24 × 10^−03^	*CTC-507E2.1*	1.68 × 10^−03^
*ZNF835*	7.36 × 10^−03^	*SMIM11*	2.09 × 10^−03^
*RP11-63E5.6*	7.54 × 10^−03^	*GJB7*	3.60 × 10^−03^
*MMP9*	7.63 × 10^−03^	*PTTG1*	4.90 × 10^−03^
*RP11-768F21.1*	8.44 × 10^−03^	*CXCL6*	5.12 × 10^−03^
*AC093668.2*	8.57 × 10^−03^	*RP11-315I20.1*	5.28 × 10^−03^
*RP11-73M18.7*	1.09 × 10^−02^	*ACOX2*	5.66 × 10^−03^
*AC061975.7*	1.14 × 10^−02^	*MAGOH2*	6.78 × 10^−03^
*AC090673.2*	1.14 × 10^−02^	*RP11-440L14.1*	6.78 × 10^−03^
*CTD-2514K5.4*	1.14 × 10^−02^	*ENPP7P10*	7.43 × 10^−03^
*MRPS21P3*	1.14 × 10^−02^	*RPL7AP30*	7.66 × 10^−03^
*RN7SL474P*	1.14 × 10^−02^	*RP11-514P8.7*	7.91 × 10^−03^

## Data Availability

RNA-sequence data were deposited in the Gene Expression Omnibus (number GSE198742).

## Supplementary Materials

The following are available online at https://www.mdpi.com/article/10.3390/ijms23126731/s1
