# Supplementary material for: Effects of Melatonin on the Transcriptome of Human Granulosa Cells, Fertilization and Blastocyst Formation
Source: Int J Mol Sci. 2022 Jun 16;23(12):6731. doi: 10.3390/ijms23126731 (PMC9223589; doi:10.3390/ijms23126731)
Supplement: Supplementary file 1 [file ijms-23-06731-s001.zip › ijms-1740707-supplementary/supplemental/Supplementa Figure S1.pptx]

## Slide 1
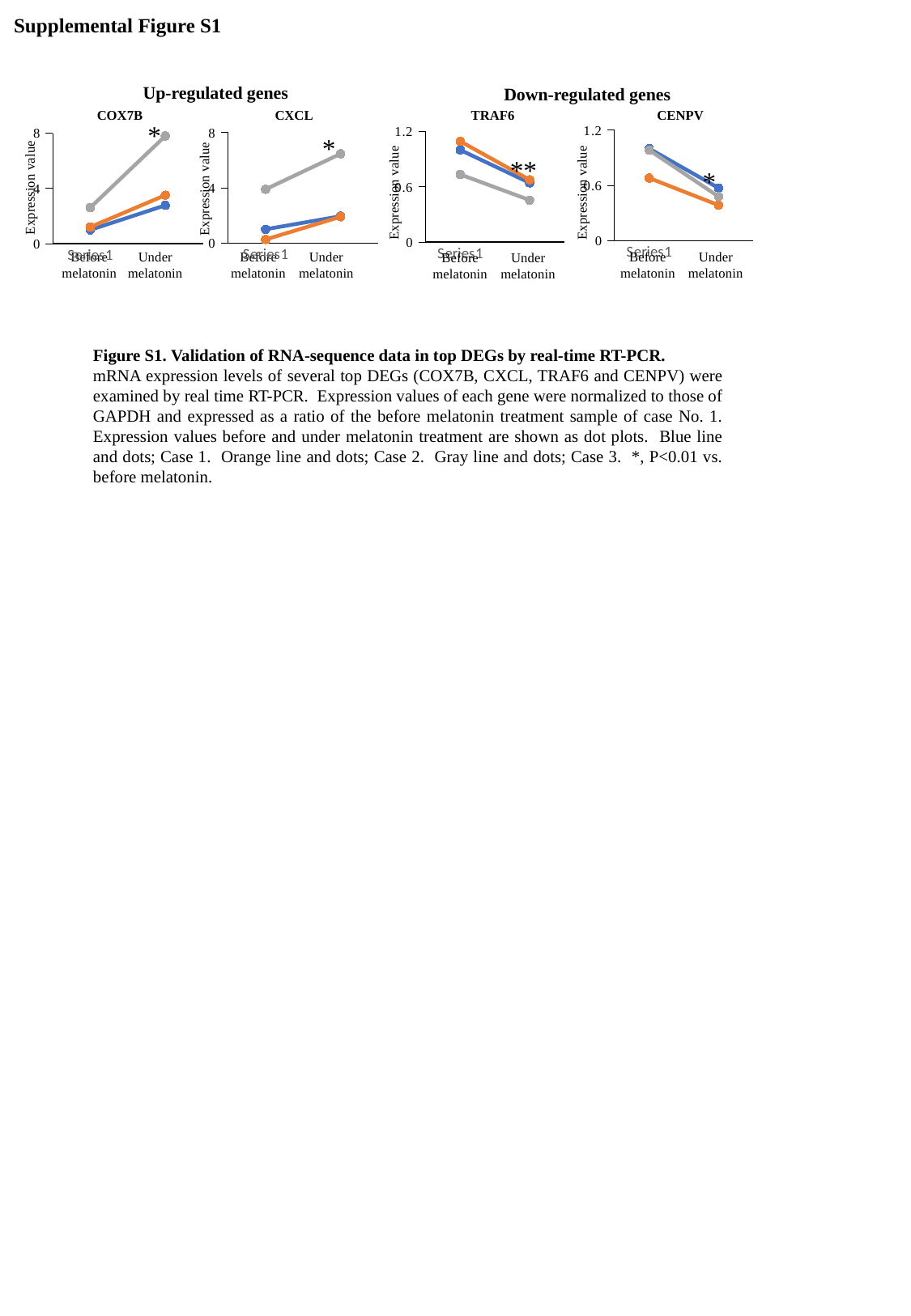

Supplemental Figure S1
Up-regulated genes
Down-regulated genes
COX7B
CXCL
TRAF6
CENPV
*
### Chart
| Category | | | |
|---|---|---|---|
| | 1.0 | 0.6806641750257068 | 0.9834860527079436 |
| | 0.5723680647707438 | 0.38555673759239095 | 0.4796320596626333 |
### Chart
| Category | | | |
|---|---|---|---|
| | 1.0 | 1.0940224446702138 | 0.7345672886122079 |
| | 0.6436784662324705 | 0.6767461055686036 | 0.4559310019990932 |
### Chart
| Category | | | |
|---|---|---|---|
| | 1.0 | 0.2633463634873656 | 3.898471310491706 |
| | 1.9479384602185457 | 1.9061286908104482 | 6.46523207135575 |
### Chart
| Category | | | |
|---|---|---|---|
| | 1.0 | 1.2095529933140134 | 2.614268854479079 |
| | 2.777587743496842 | 3.5127559716661625 | 7.797079421606275 |*
**
*
Expression value
Expression value
Expression value
Expression value
Before
melatonin
Under
melatonin
Before
melatonin
Under
melatonin
Before
melatonin
Under
melatonin
Before
melatonin
Under
melatonin
Figure S1. Validation of RNA-sequence data in top DEGs by real-time RT-PCR.
mRNA expression levels of several top DEGs (COX7B, CXCL, TRAF6 and CENPV) were examined by real time RT-PCR. Expression values of each gene were normalized to those of GAPDH and expressed as a ratio of the before melatonin treatment sample of case No. 1. Expression values before and under melatonin treatment are shown as dot plots. Blue line and dots; Case 1. Orange line and dots; Case 2. Gray line and dots; Case 3. *, P<0.01 vs. before melatonin.
